# Association of anemia with diabetic kidney disease in adults with type 2 diabetes: A cross-sectional NHANES study and 2-sample Mendelian randomization analysis

**DOI:** 10.1097/MD.0000000000050065

**Published:** 2026-08-07

**Authors:** Xi Sun, Huanjing Wang, Zhanzheng Wang, Yanyun Ren

**Affiliations:** a Department of Nephrology, Affiliated Hospital of Shaanxi University of Traditional Chinese Medicine, Shaanxi, China; b Department of Clinical Laboratory, Affiliated Hospital of Shaanxi University of Traditional Chinese Medicine, Shaanxi, China.

**Keywords:** anemia, cross-sectional study, diabetic kidney disease, hemoglobin, Mendelian randomization, NHANES

## Abstract

Anemia commonly accompanies chronic kidney disease, but the direction of the association between anemia and diabetic kidney disease remains uncertain. We examined the association using cross-sectional NHANES data and explored potential causal associations using 2-sample Mendelian randomization. We conducted a cross-sectional analysis of adults with type 2 diabetes from the 2009–2018 NHANES cycles. Survey-weighted logistic regression and restricted cubic spline analyses were used to evaluate the associations of anemia and hemoglobin levels with presumed DKD, albuminuria, and reduced eGFR. Two-sample Mendelian randomization was subsequently performed to explore potential causal associations between anemia-related phenotypes and DKD. Anemia was more prevalent among participants with presumed DKD than among those without presumed DKD (21.6% vs 9.6%). In survey-weighted regression models, anemia was associated with higher odds of presumed DKD, albuminuria, and reduced eGFR, whereas higher hemoglobin levels were inversely associated with these outcomes. Restricted cubic spline analyses indicated L-shaped associations. Mendelian randomization estimates suggested potential associations of genetically predicted general anemia, iron deficiency anemia, and vitamin B12 deficiency anemia with DKD; however, significant heterogeneity was observed across SNP-specific estimates. Anemia was associated with higher odds of presumed DKD, albuminuria, and reduced eGFR among adults with type 2 diabetes. Mendelian randomization analyses provided suggestive evidence of potential causal associations between anemia-related phenotypes and DKD. However, the cross-sectional design, relaxed SNP-selection threshold, and significant heterogeneity warrant cautious interpretation and independent validation.

## 1. Introduction

Diabetes has been identified as a significant public health concern globally, with incidence rates rising consistently across the world. Diabetic kidney disease (DKD), a microvascular complication of diabetes and a form of chronic kidney disease (CKD) attributable to diabetes mellitus (DM), has a multifaceted etiology. Clinically, DKD is characterized by persistent albuminuria and a gradual reduction in the glomerular filtration rate (GFR), ultimately leading to end-stage renal disease (ESRD). Presently, DKD is recognized as the most frequent precursor to ESRD and renal replacement therapy, affecting approximately 30% of patients with type 1 diabetes and 40% of those with type 2 diabetes. The impact of DKD extends beyond public health concerns, significantly impeding socioeconomic progress. Consequently, early detection of modifiable risk factors for DKD is crucial for the prevention or postponement of kidney function deterioration.

Anemia is a common clinical manifestation among patients with CKD. It serves as both an important complication of kidney disease and a frequent comorbid condition.^[1,2]^ Among patients with CKD, those with DKD are more prone to developing anemia than those with nondiabetic kidney disease (NDKD).^[3]^ The development of anemia in DKD patients is multifactorial and involves various factors, such as the progression of kidney disease, inflammation, nutritional deficiencies, coexisting autoimmune disorders, medications, and hormonal changes.^[4]^ Thyroid dysfunction can also contribute to anemia in DKD patients.^[5,6]^ Compared with nondiabetic individuals, diabetic patients present a greater prevalence and severity of anemia at each stage of kidney disease.^[7]^ Anemia has been associated with faster DKD progression and a higher risk of cardiovascular disease, diabetic retinopathy, and diabetic neuropathy. Additionally, anemia can lead to spuriously low glycated hemoglobin levels (HbA1c), potentially resulting in inadequate management of hyperglycemia and further exacerbating the development of microvascular and macrovascular complications in patients with diabetes.^[8]^ Increasing evidence suggests that anemia is associated with renal impairment and may also be involved in diabetic kidney injury. The RENAAL study reported that hemoglobin levels are an independent risk factor for the progression of diabetic kidney disease.^[9]^ Another study demonstrated an independent correlation between the severity of albuminuria and the increased incidence of anemia after adjusting for renal function.^[10]^ Nevertheless, because anemia is also a recognized consequence of impaired kidney function, the direction and independence of the association between anemia and DKD remain uncertain.

Studies directly examining the associations between anemia/hemoglobin levels and DKD, urinary albumin-to-creatinine ratio (UACR), and low eGFR are limited. Further large-scale studies are needed to confirm the relationships between anemia/hemoglobin levels and DKD, UACR, and low GFR. Additionally, research is needed to evaluate the predictive value of anemia/hemoglobin levels in DKD progression. In this study, we used cross-sectional data from the National Health and Nutrition Examination Survey (NHANES) to evaluate the associations of anemia and hemoglobin levels with presumed DKD, albuminuria, and reduced eGFR after adjustment for relevant demographic, behavioral, and clinical covariates. Mendelian randomization (MR) is a genetic epidemiological approach that uses genetic variants as instrumental variables to investigate potential causal associations between exposures and outcomes,^[11]^ and offers inherent advantages, being less susceptible to confounding factors.^[12]^ To complement the cross-sectional analysis and explore whether the observed association might reflect a potential causal relationship, we performed a 2-sample Mendelian randomization analysis. Given the inherent limitations of both the observational and genetic analyses, the findings were interpreted as evidence of association and potential causality rather than definitive proof of a causal effect.

This study aimed to evaluate the associations of anemia and hemoglobin levels with presumed DKD, albuminuria, and reduced eGFR in adults with type 2 diabetes and to explore potential causal associations using 2-sample Mendelian randomization.

## 2. Materials and methods

### 2.1. Observational study of NHANES

**Figure 1.**
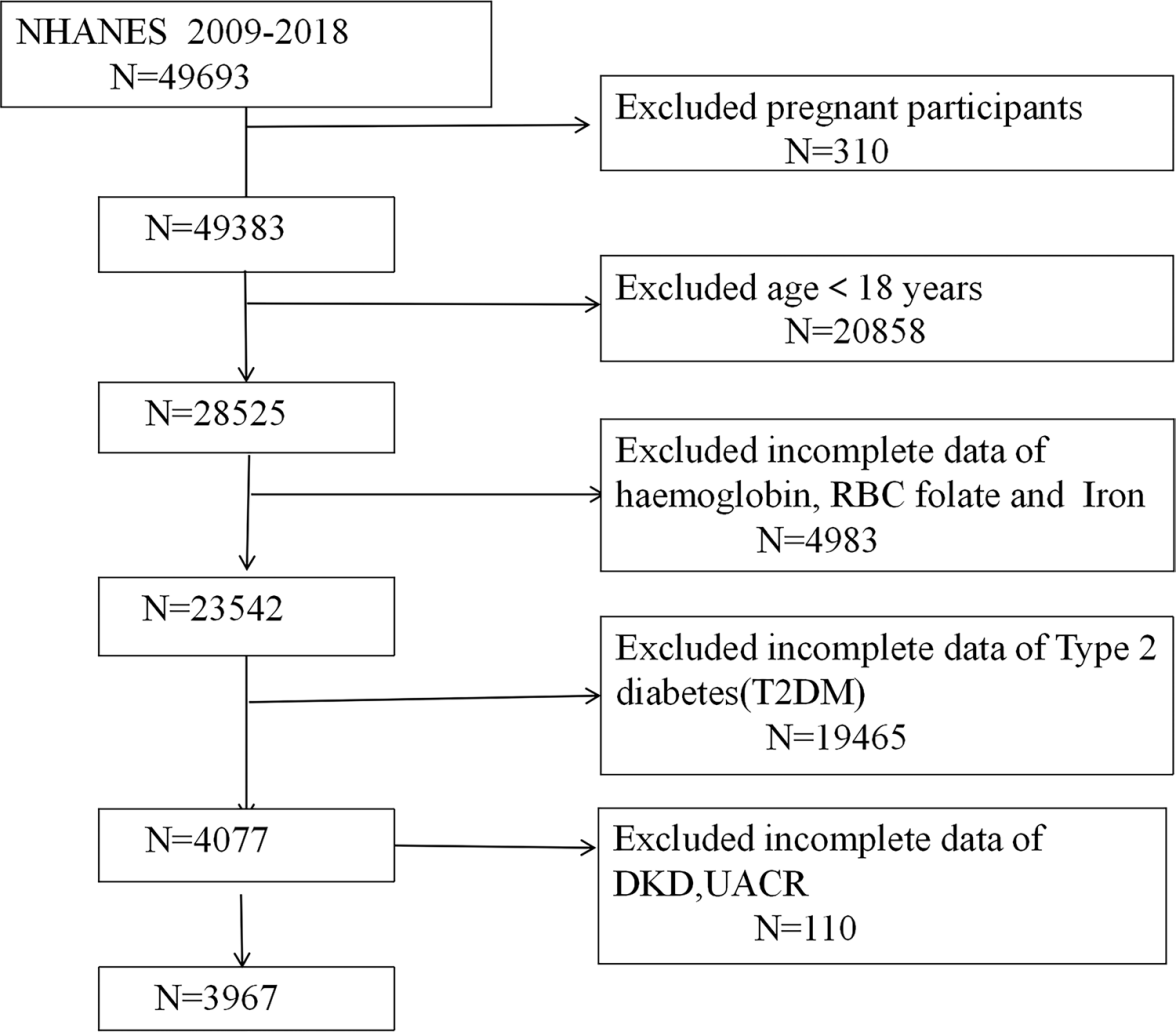
Flow diagram of this study. DKD = diabetic kidney disease, NHANES = National Health and Nutrition Examination Survey, UACR = urinary albumin-to-creatinine ratio.

The NHANES offers a cross-sectional perspective on the health of the US population,^[13]^ and evaluates the nutritional and physical health of the noninstitutionalized population. Detailed information on NHANES is available at http://www.cdc.gov/nchs/nhanes.htm. We analyzed data from adults aged ≥18 years who participated in the 2009–2018 NHANES cycles. Laboratory measurements included hemoglobin, fasting blood glucose, glycated hemoglobin (HbA1c), urinary albumin-to-creatinine ratio (UACR), and serum creatinine. Questionnaire data included self-reported diabetes history and the use of glucose-lowering medications or insulin. Demographic and clinical variables included age, gender, race/ethnicity, educational level, poverty-to-income ratio (PIR), smoking status, alcohol consumption, body mass index (BMI), and hypertension status. Participants were excluded if they were pregnant, did not meet the criteria for type 2 diabetes, or had missing data on hemoglobin, serum creatinine, or UACR. A total of 3967 participants were included in the final analysis (Fig. 1). This study was reported in accordance with the Strengthening the Reporting of Observational Studies in Epidemiology (STROBE) guidelines.

#### 2.1.1. Diagnosis of DKD

In this study, participants meeting any of these criteria were classified as having diabetes^[14]^: a physician’s diagnosis, use of glucose-lowering medications or insulin, or fasting blood glucose levels exceeding 7.0 mmol/L (126 mg/dL) or HbA1c levels exceeding 6.5%. For the cross-sectional NHANES analysis, participants with type 2 diabetes who had a UACR ≥ 30 mg/g and/or an eGFR < 60 mL/min/1.73 m^2^ were classified as having presumed DKD. Because NHANES provides measurements from a single examination cycle, persistence of albuminuria or reduced eGFR for at least 3 months could not be confirmed. In addition, kidney disease attributable to causes other than diabetes could not be completely excluded.^[15,16]^

#### 2.1.2. Diagnosis of anemia

Anemia was defined according to the diagnostic criteria of the World Health Organization, with serum hemoglobin levels < 13 g/dL for adult males and <12 g/dL for adult females considered anemic.^[17]^

#### 2.1.3. Covariates

The covariates in this study included age, gender, race/ethnicity (Mexican-American, Non-Hispanic White, Non-Hispanic Black, other races), BMI, smoking status, alcohol consumption, education level (below high school and high school or above), PIR, and hypertension. A total of 3967 participants were included in the final analysis, comprising 2408 participants without presumed DKD and 1559 participants with presumed DKD.

#### 2.1.4. Statistical analysis

On the basis of the recommendations of the US Centers for Disease Control and Prevention (CDC), all the statistical analyses in this study considered the complex sample design of the multistage cluster survey.^[18]^ The NHANES database release files provide weights for each sample. Owing to the inclusion of a large amount of laboratory data obtained at the Mobile Examination Center (MEC), we utilized the combined wtmec2yr weight for weighted analysis. For continuous variables, the means ± standard deviations (SDs) are reported, whereas categorical variables are presented as individual counts (N) and percentages (%). To evaluate differences between groups, weighted *t* tests were used for continuous variables, and chi-square tests were used for categorical variables. Weighted logistic regression analysis and restricted cubic spline (RCS) analysis were used to investigate the associations and correlations between hemoglobin levels and DKD, albuminuria, and low eGFR. A complex sampling, multi-factor logistic regression model was constructed to evaluate the relationships between anemia and clinical variables after adjusting for multiple factors. All the statistical analyses were conducted via R software version 4.3.1 (R Foundation for Statistical Computing). A significance level of *P* < .05 was considered statistically significant for all the statistical analyses.

### 2.2. Mendelian randomization analysis

#### 2.2.1. Study design

**Figure 2.**
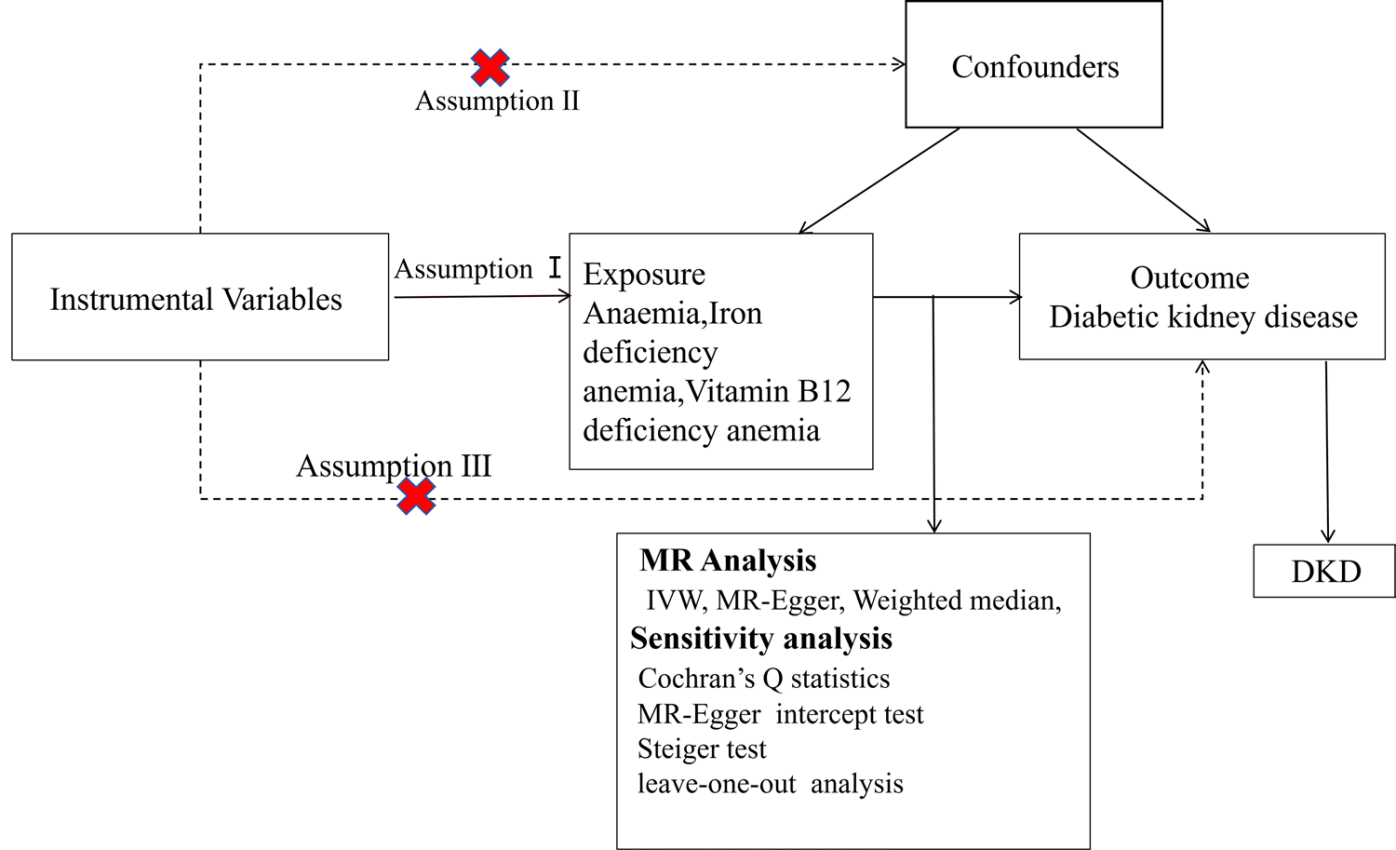
Mendelian randomization model and Mendelian randomization analysis. DKD = diabetic kidney disease, IVW = inverse-variance weighted, MR = Mendelian randomization.

This study primarily conducted a 2-sample MR analysis to explore the causal association between anemia and DKD. This analysis followed 3 core assumptions^[19]^: the instrumental variable is significantly associated with the exposure; the instrumental variable is independent of any potential confounding factors; and genetic variation influences the outcome solely through the exposure and not through any other pathways. Ethics approval was not required for our study, as all the data used were derived from publicly available aggregated statistical data (Fig. 2).

#### 2.2.2. Data sources

Comprehensive genome-wide association study (GWAS) data related to anemia and DKD were obtained from the FinnGen database (https://www.finngen.fi/en/access_results). Details of the GWASs included in the Mendelian randomization are shown in Table 1.

**Table 1:** Details of the GWAS studies included in the Mendelian randomization.

Phenotypes	GWAS ID	Consortium	Year	Sample size	Case	Control	SNPs	Ancestry
Anemia	Finngen R10 D3 ANEMIA	FinnGen	2023	1,24,542	30,092	94,450	19321023	European
Iron deficiency anemia	Finngen R10 D3 ANEMIA IRONDEF	FinnGen	2023	4,08,837	15,153	3,93,684	19345589	European
Vitamin B_12_ deficiency anemia	Finngen R10 D3 ANEMIA B12 DEF	FinnGen	2023	3,97,378	3694	3,93,684	19345434	European
DKD	Finngen_R9_DM_NEPHROPATHY_EXMORE	FinnGen	2023	3,12,650	4111	3,08,539	NA	European

DKD = diabetic kidney disease, GWAS = genome-wide association study, SNP= single-nucleotide polymorphism.

#### 2.2.3. Selection of IVs

This study used broad anemia, iron deficiency anemia, and vitamin B_12_ deficiency anemia as exposure variables and single nucleotide polymorphisms (SNPs) associated with anemia as instrumental variables (IVs) to investigate their causal relationship with DKD as the outcome variable. Because only a limited number of independent variants reached the conventional genome-wide significance threshold of *P* < 5 × 10^−8^ for some anemia phenotypes, a relaxed threshold of *P* < 5 × 10^−6^ was applied to increase instrument availability. Independent variants were selected using an LD threshold of *r*^2^ < 0.001 within a 10,000-kb window. Harmonization was performed to exclude incompatible variants and palindromic variants with ambiguous allele frequencies. The use of the relaxed significance threshold may increase susceptibility to weak-instrument bias; therefore, the MR findings were interpreted cautiously.

#### 2.2.4. Statistical analysis

The inverse-variance weighted (IVW) method was used as the primary estimator to evaluate potential causal associations between anemia-related phenotypes and DKD. Weighted-median and MR-Egger analyses were performed as complementary methods. Heterogeneity was assessed using Cochran *Q* test, and horizontal pleiotropy was evaluated using the MR-Egger intercept test. Leave-one-out analyses were conducted to assess whether any single SNP disproportionately influenced the pooled estimate. In addition, the MR Steiger test was used to evaluate the direction of the exposure–outcome association. All analyses were conducted using R software version 4.3.1. A 2-sided *P* value < .05 was considered statistically significant. The results were interpreted as suggestive evidence of potential causality rather than definitive causal proof.

## 3. Results

### 3.1. Cross-sectional study

#### 3.1.1. Basic characteristics of the study participants

A total of 49,693 participants from the 2009–2018 NHANES cycles were initially considered. After applying the eligibility criteria, 3967 adults with type 2 diabetes were included in the final analysis, of whom 1559 were classified as having presumed DKD. The weighted prevalence of presumed DKD was 35.48%. Participants with presumed DKD had a lower mean Hb concentration than those without presumed DKD (13.69 vs 14.18 g/dL), whereas the prevalence of anemia was higher (21.6% vs 9.6%; both *P* < .001). Compared with participants without presumed DKD, those with presumed DKD were older and had a higher prevalence of hypertension, but lower proportions of smoking and alcohol consumption. They also had lower red blood cell (RBC) counts, eGFR, serum albumin, and serum iron levels, but higher mean corpuscular volume, RBC folate, serum folate, UACR, serum creatinine, blood urea nitrogen, uric acid, HbA1c, triglyceride, and fasting blood glucose levels. The corresponding P values are presented in Table 2.

**Table 2:** Clinical characteristics of participants with type 2 diabetes with or without presumed DKD.

Characteristic	Without DKD	DKD	*P*-value
Unweighted number	N = 2408	N = 1559	
Weighted number	N = 1,59,06,173	N = 87,48,551	
Age (yr)	55.68 (12.93)	64.28 (13.21)	<.001
Gender (%)			.637
Female	74,63,758 (46.9)	42,16,386 (48.2)	
Male	84,42,414 (53.1)	45,32,165 (51.8)	
Anemia (%)	1524,123 (9.6)	1891,873 (21.6)	<.001
Hb (g/dL)	14.18 (1.44)	13.69 (1.75)	<.001
RBC (million cells/μL)	4.74 (0.46)	4.56 (0.61)	<.001
MCV (fL)	88.68 (5.33)	89.42 (6.11)	.006
Iron (μg/dL)	79.71 (31.14)	75.86 (29.49)	.008
RBC folate (nmol/L)	1327.59 (589.63)	1510.70 (798.22)	<.001
Serum folate (nmol/L)	20.40 (12.93)	23.75 (27.78)	.017
UACR (mg/g)	10.01 (6.27)	323.25 (956.13)	<.001
Creatinine (mg/dL)	0.82 (0.18)	1.21 (0.82)	<.001
BUN (mmol/L)	4.95 (1.63)	7.05 (3.60)	<.001
eGFR (ml/min/1.73 m^2^)	93.28 (17.04)	68.32 (28.46)	<.001
Uric acid (μmol/L)	322.81 (78.74)	371.00 (101.55)	<.001
Triglyceride (mmol/L)	1.72 (1.18)	1.95 (1.73)	.002
HbA1c (%)	7.11 (1.52)	7.57 (1.86)	<.001
HDL (mmol/L)	1.22 (0.35)	1.22 (0.45)	.801
LDL (mmol/L)	2.74 (0.93)	2.67 (1.05)	.271
FBG (mmol/L)	8.40 (2.87)	9.13 (3.56)	<.001
BMI (kg/m^2^)	33.20 (7.46)	33.22 (7.45)	.931
Albumin (g/L)	41.91 (3.28)	40.75 (3.57)	<.001
Race (%)			.338
Mexican American	17,24,348 (10.8)	9,25,709 (10.6)	
Non-Hispanic Black	93,96,051 (59.1)	52,08,849 (59.5)	
Non-Hispanic White	21,85,194 (13.7)	13,23,991 (15.1)	
Other	26,00,579 (16.3)	12,90,000 (14.7)	
Education (%)			.002
Below high school	33,98,611 (21.4)	23,10,690 (26.4)	
High School or above	1,25,07,562 (78.6)	64,37,861 (73.6)	
PIR (%)			.004
Not poor	1,35,84,743 (85.4)	70,93,373 (81.1)	
Poor	64,37,861 (14.6)	16,55,178 (18.9)	
Smoke (%)			.011
Yes	27,47,584 (17.3)	11,83,935 (13.5)	
No	1,31,58,589 (82.7)	75,64,616 (86.5)	
Drink (%)			<.001
Yes	1,12,17,417 (75.4)	55,29,400 (66.9)	
No	36,58,014 (24.6)	27,40,268 (33.1)	
Hypertension			<.001
Yes	94,47,235 (59.4)	64,52,203 (73.8)	
No	64,58,938 (40.6)	22,96,348 (26.2)	

BMI = body mass index, BUN = blood urea nitrogen, DKD = diabetic kidney disease, eGFR = estimated glomerular filtration rate, FBG = fasting blood glucose, HDL = high-density lipoprotein cholesterol, LDL = low-density lipoprotein cholesterol, MCV = mean corpuscular volume, PIR = poverty-to-income ratio, RBC = red blood cell, UACR = urinary albumin-to-creatinine ratio.

#### 3.1.2. Associations of anemia with clinical characteristics among participants with type 2 diabetes

Among the 3967 participants with type 2 diabetes, 729 had anemia, corresponding to a weighted anemia prevalence of 13.86%. Anemic participants had a higher prevalence of presumed DKD than nonanemic participants (55.4% vs 32.3%). Compared with nonanemic participants, those with anemia had lower Hb, RBC count, mean corpuscular volume, serum iron, eGFR, low-density lipoprotein cholesterol, serum albumin, and fasting blood glucose levels, but higher RBC folate, serum folate, UACR, serum creatinine, blood urea nitrogen, and uric acid levels. Anemic participants also had higher proportions of UACR ≥ 30 mg/g, reduced eGFR, and hypertension. The corresponding *P* values are presented in Table 3.

**Table 3:** Clinical characteristics of participants with type 2 diabetes according to anemia status.

Characteristic	No anemia	Anemia	*P*-value
Unweighted number	N = 3238	N = 729	
Weighted number	N = 2,12,38,728	N = 34,15,996	
Age (yr)	58.07 (13.48)	62.81 (14.08)	<.001
Gender (%)			.026
Female	1,14,65,581 (54.0)	15,08,999 (44.2)	
Male	97,73,147 (46.0)	19,06,997 (55.8)	
DKD (%)	68,56,678 (32.3)	18,91,873 (55.4)	<.001
Hb (g/dL)	14.41 (1.24)	11.50 (0.99)	<.001
RBC (million cells/μL)	4.78 (0.45)	4.05 (0.48)	<.001
MCV (fL)	89.35 (5.00)	86.38 (8.12)	<.001
Iron (μg/dL)	81.53 (29.51)	58.54 (29.93)	<.001
RBC folate (nmol/L)	1369.92 (646.99)	1533.33 (824.90)	<.001
Serum folate (nmol/L)	21.27 (19.48)	23.57 (20.17)	.045
UACR (mg/g)	89.50 (453.41)	318.01 (1086.70)	<.001
UACR ≥ 30 mg/g (%)	49,35,909.7 (23.2)	13,71,694.0 (40.2)	<.001
Creatinine (mg/dL)	0.91 (0.34)	1.30 (1.13)	<.001
BUN (mmol/L)	5.43 (2.25)	7.35 (4.26)	<.001
eGFR (mL/min/1.73 m^2^)	86.76 (23.00)	69.88 (30.34)	<.001
eGFR ≤ 60mL/min/1.73 m^2^	30,95,442.0 (14.6)	13,01,861.2 (38.1)	<.001
Uric acid (μmol/L)	337.86 (88.54)	352.72 (100.97)	.010
HbA1c (%)	7.29 (1.68)	7.12 (1.53)	<.001
Triglyceride (mmol/L)	1.84 (1.45)	1.51 (0.99)	<.001
HDL (mmol/L)	1.21 (0.39)	1.28 (0.39)	.001
LDL (mmol/L)	2.75 (0.98)	2.48 (0.87)	<.001
FBG (mmol/L)	8.76 (3.18)	7.87 (2.81)	<.001
BMI (kg/m^2^)	33.31 (7.40)	32.57 (7.78)	.080
Albumin (g/L)	41.81 (3.28)	39.59 (3.72)	<.001
Race (%)			<.001
Mexican American	22,80,760 (10.7)	3,69,297 (10.8)	
Non-Hispanic Black	1,30,11,915 (61.3)	15,92,984 (46.6)	
Non-Hispanic White	25,94,038 (12.2)	9,15,148 (26.8)	
Other	33,52,014 (15.8)	5,38,566 (15.8)	
Education (%)			.011
Below high school	47,31,681 (22.3)	9,77,620 (28.6)	
High School or above	1,65,07,047 (77.7)	24,38,376 (71.4)	
PIR (%)			.086
Not poor	1,78,98,676 (84.3)	27,79,439 (81.4)	
Poor	33,40,051 (15.7)	6,36,557 (18.6)	
Smoke (%)			<.001
Yes	36,58,124 (17.2)	2,73,395 (8.0)	
No	1,75,80,604 (82.8)	31,42,601 (92.0)	
Drink (%)			<.001
Yes	1,47,51,832 (73.8)	19,94,985 (63.3)	
No	52,41,792 (26.2)	11,56,491 (36.7)	
Hypertension			.006
Yes	1,34,53,232 (63.3)	24,46,206 (71.6)	
No	77,85,496 (36.7)	9,69,790 (28.4)	

BMI = body mass index, BUN = blood urea nitrogen, DKD = diabetic kidney disease, eGFR = estimated glomerular filtration rate, FBG = fasting blood glucose, HDL = high-density lipoprotein cholesterol, LDL = low-density lipoprotein cholesterol, MCV = mean corpuscular volume, PIR = poverty-to-income ratio, RBC = red blood cell, UACR = urinary albumin-to-creatinine ratio.

Survey-weighted multivariable logistic regression analysis was performed to evaluate the associations of clinical parameters with the odds of anemia. After adjustment for age, gender, and race/ethnicity, higher UACR, blood urea nitrogen, and uric acid levels, together with lower eGFR, were associated with higher odds of anemia. The corresponding *P* values were .044, .001, .027, and .003, respectively (Table 4).

**Table 4:** Logistic analysis of anemia and the clinical characteristics of diabetic patients.

Variable	β	SE	*t*	*P*	OR	95% CI	
UACR	0.00	0.00	2.04	.044	1.00	0.00	0.00
eGFR	−0.01	0.00	−3.06	.003	0.99	−0.02	−0.01
Uric acid	−0.00	0.00	−2.25	.027	1.00	−0.00	−0.00
BUN	0.10	0.03	3.87	.001	1.11	0.05	0.16
Creatinine	0.32	0.18	1.79	.078	1.38	0.00	0.68
HbA1c	−0.07	0.04	−1.69	.095	0.94	−0.15	0.01

BUN = blood urea nitrogen, CI = confidence interval, eGFR = estimated glomerular filtration rate, OR = odds ratio, SE = standard error, UACR = urinary albumin-to-creatinine ratio.

#### 3.1.3. Association between anemia/hemoglobin (Hb) and DKD

**Figure 3.**
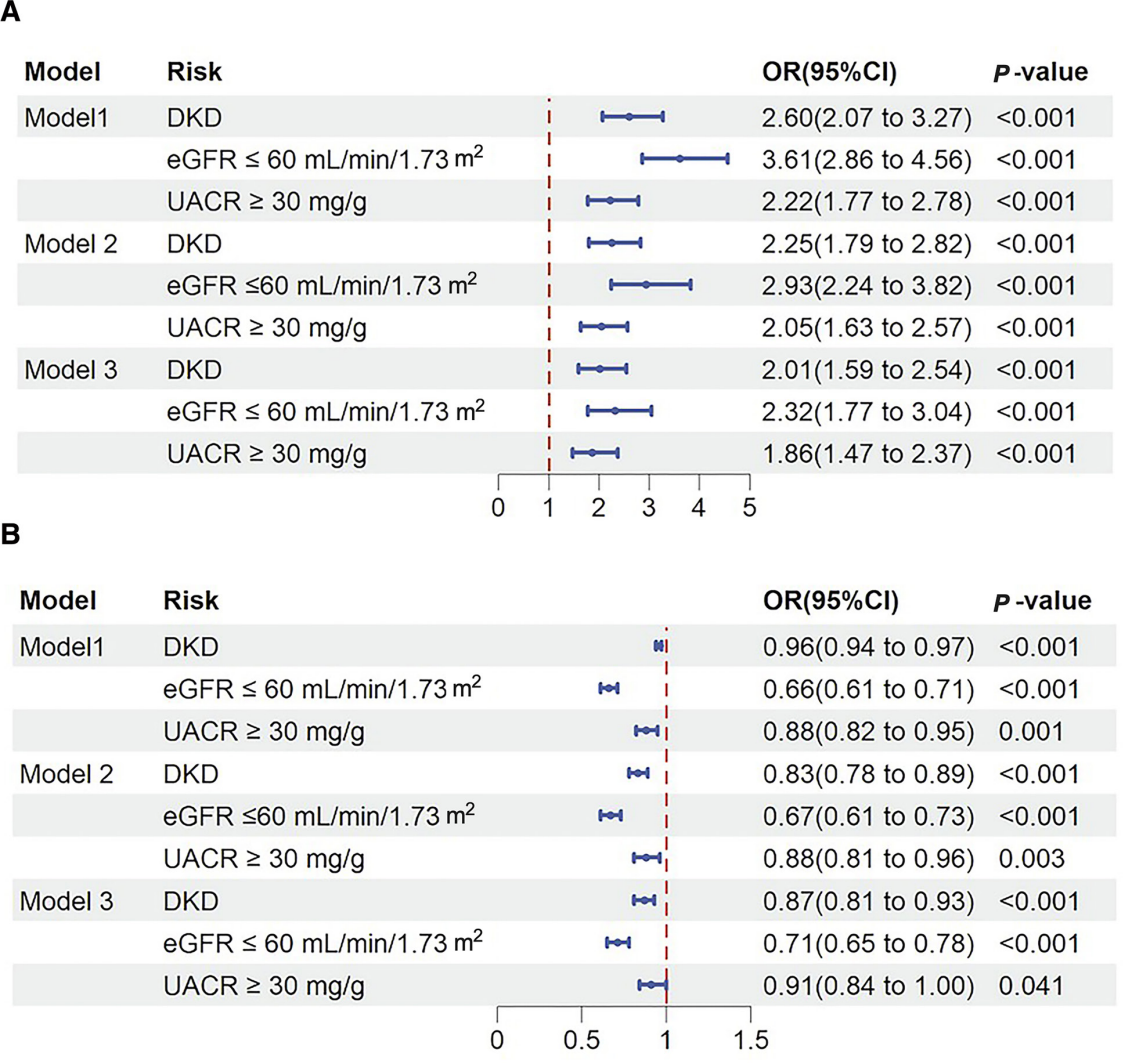
Associations of anemia and hemoglobin levels with presumed DKD, reduced eGFR, and albuminuria. (A) Associations of anemia with the odds of presumed DKD, reduced eGFR, and UACR ≥ 30 mg/g. (B) Associations of hemoglobin levels with these outcomes. Model 1 was unadjusted; model 2 was adjusted for age and gender; and model 3 was additionally adjusted for HbA1c, blood pressure, and serum albumin. CI = confidence interval, DKD = diabetic kidney disease, eGFR = estimated glomerular filtration rate, OR = odds ratio, UACR = urinary albumin-to-creatinine ratio.

To examine the associations of anemia and hemoglobin (Hb) levels with presumed DKD, albuminuria, and reduced eGFR, 3 weighted logistic regression models were constructed. Model 1 was unadjusted; model 2 was adjusted for age and gender; and model 3 was further adjusted for HbA1c, blood pressure, and serum albumin. As shown in Figure 3A, anemia was significantly associated with higher odds of presumed DKD, UACR ≥ 30 mg/g, and reduced eGFR in all 3 models. In contrast, higher Hb levels were significantly associated with lower odds of presumed DKD, UACR ≥ 30 mg/g, and reduced eGFR in all 3 models (Fig. 3B).

**Figure 4.**
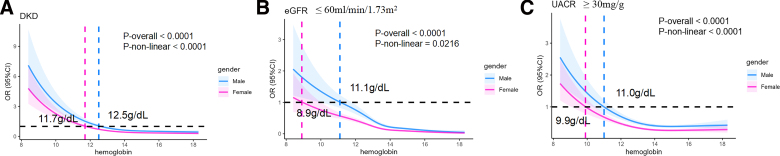
Restricted cubic spline analyses of the associations between hemoglobin levels and presumed DKD, reduced eGFR, and albuminuria. (A) Association between hemoglobin levels and the odds of presumed DKD. (B) Association between hemoglobin levels and the odds of reduced eGFR. (C) Association between hemoglobin levels and the odds of UACR ≥ 30 mg/g. CI = confidence interval, DKD = diabetic kidney disease, eGFR = estimated glomerular filtration rate, OR = odds ratio, UACR = urinary albumin-to-creatinine ratio.

To further characterize these associations, restricted cubic spline analyses were performed. As shown in Figure 4, nonlinear associations were observed between Hb levels and the odds of presumed DKD, UACR ≥ 30 mg/g, and reduced eGFR. In both men and women with type 2 diabetes, the estimated odds of these outcomes generally decreased as Hb levels increased, followed by a gradual attenuation of the associations at higher Hb levels.

Specifically, the estimated turning points for the association between Hb levels and presumed DKD were approximately 12.5 g/dL in men and 11.7 g/dL in women. Below these values, increasing Hb levels were associated with a marked reduction in the estimated odds of presumed DKD, whereas above these values, the decline in the estimated odds became less pronounced (Fig. 4A). The corresponding turning points for reduced eGFR were approximately 11.1 g/dL in men and 8.9 g/dL in women. Below these values, higher Hb levels were associated with lower odds of reduced eGFR, while the association gradually attenuated above these values (Fig. 4B). Similarly, the estimated turning points for UACR ≥ 30 mg/g were approximately 11.0 g/dL in men and 9.9 g/dL in women. Below these values, the estimated odds of UACR ≥ 30 mg/g decreased markedly with increasing Hb levels, whereas the decline became less pronounced above these values (Fig. 4C).

### 3.2. MR study

#### 3.2.1. Selection of genetic IVs for MR

**Figure 5.**
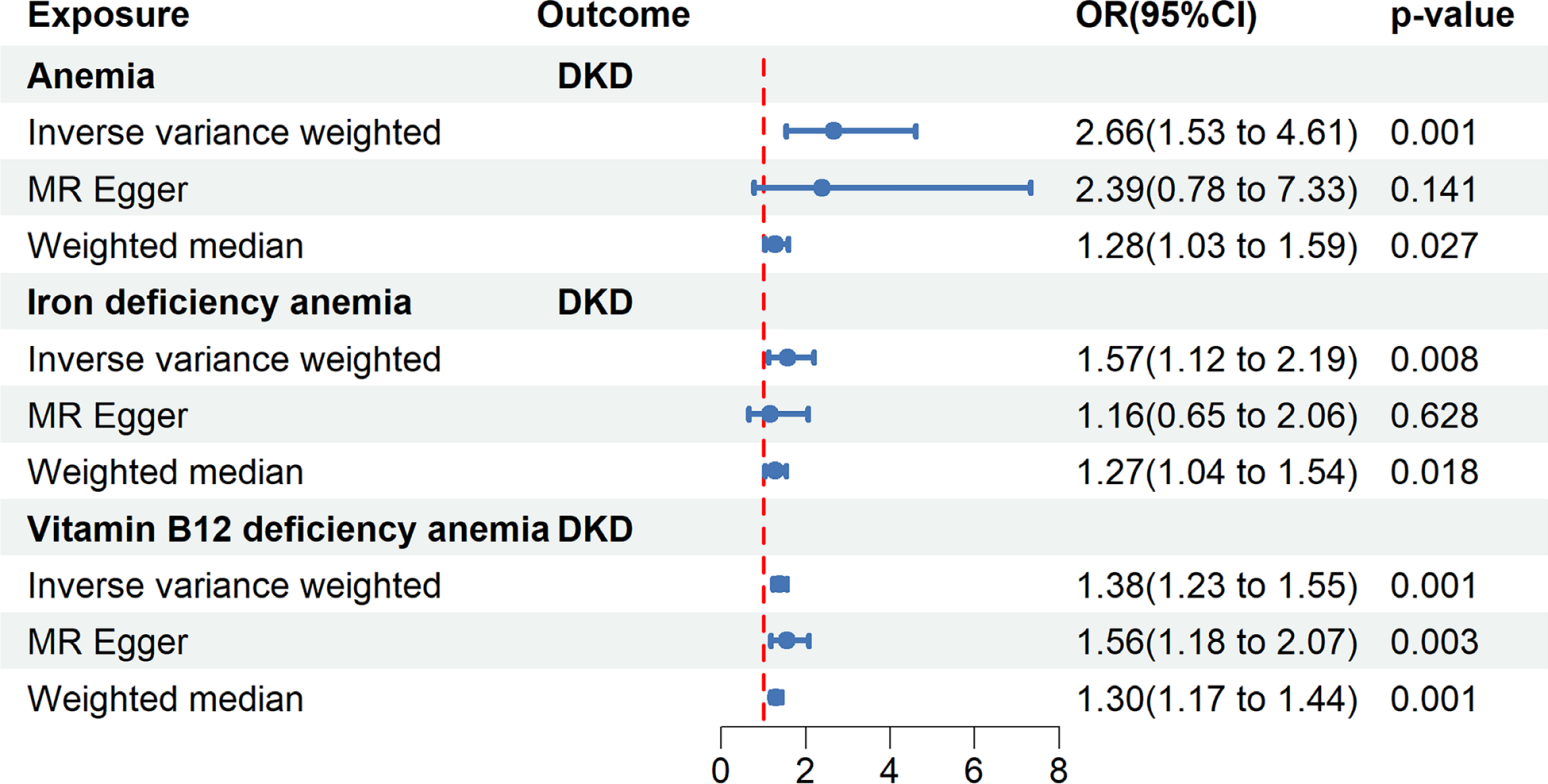
Mendelian randomization estimates for the associations of anemia-related phenotypes with DKD using IVW, MR-Egger, and weighted-median methods. CI = single-nucleotide polymorphism, DKD = diabetic kidney disease, IVW = inverse-variance weighted, MR = Mendelian randomization, OR = odds ratio.

**Figure 6.**
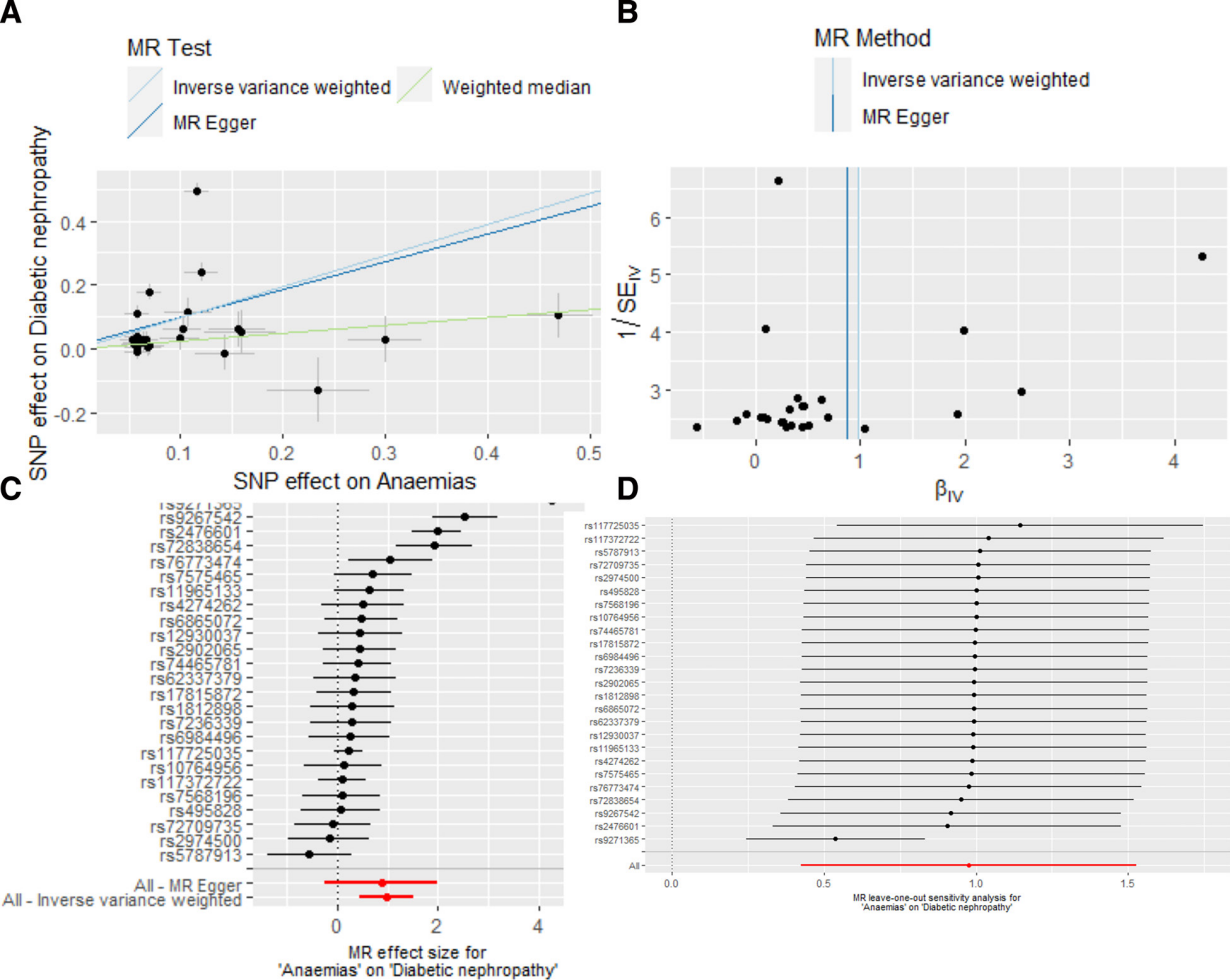
Mendelian randomization analyses of the association between general anemia and DKD. (A) Scatter plot of SNP-specific associations. (B) Funnel plot used to assess asymmetry. (C) Forest plot of SNP-specific and pooled MR estimates. (D) Leave-one-out analysis used to assess whether any single SNP disproportionately influenced the pooled estimate. DKD = diabetic kidney disease, IVW = inverse-variance weighted, MR = Mendelian randomization, SE = standard error, SNP = single-nucleotide polymorphism.

**Figure 7.**
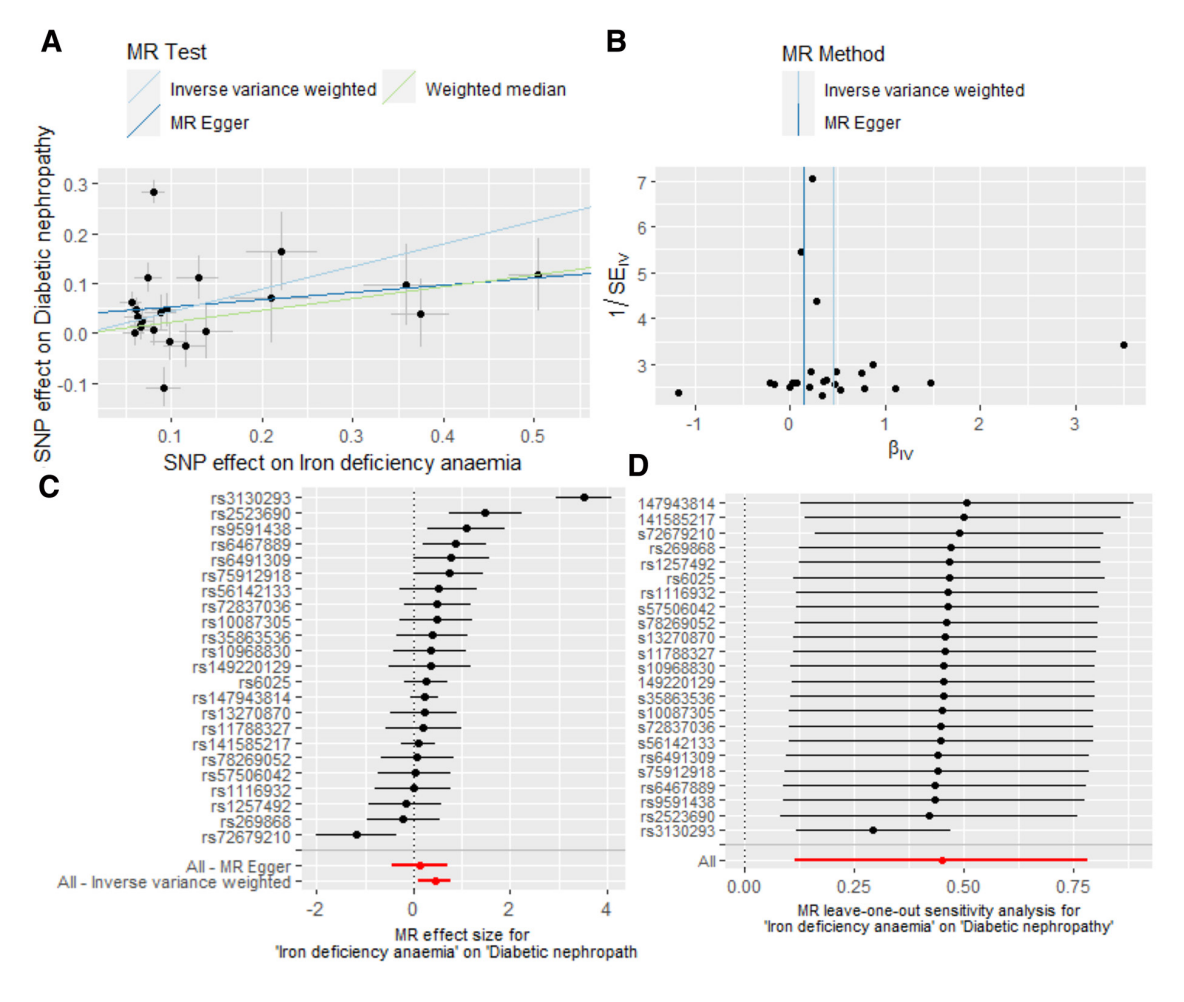
Mendelian randomization analyses of the association between iron deficiency anemia and DKD. (A) Scatter plot of SNP-specific associations. (B) Funnel plot used to assess asymmetry. (C) Forest plot of SNP-specific and pooled MR estimates. (D) Leave-one-out analysis used to assess whether any single SNP disproportionately influenced the pooled estimate. DKD = diabetic kidney disease, IVW = inverse-variance weighted, MR = Mendelian randomization, SE = standard error, SNP= single-nucleotide polymorphism.

**Figure 8.**
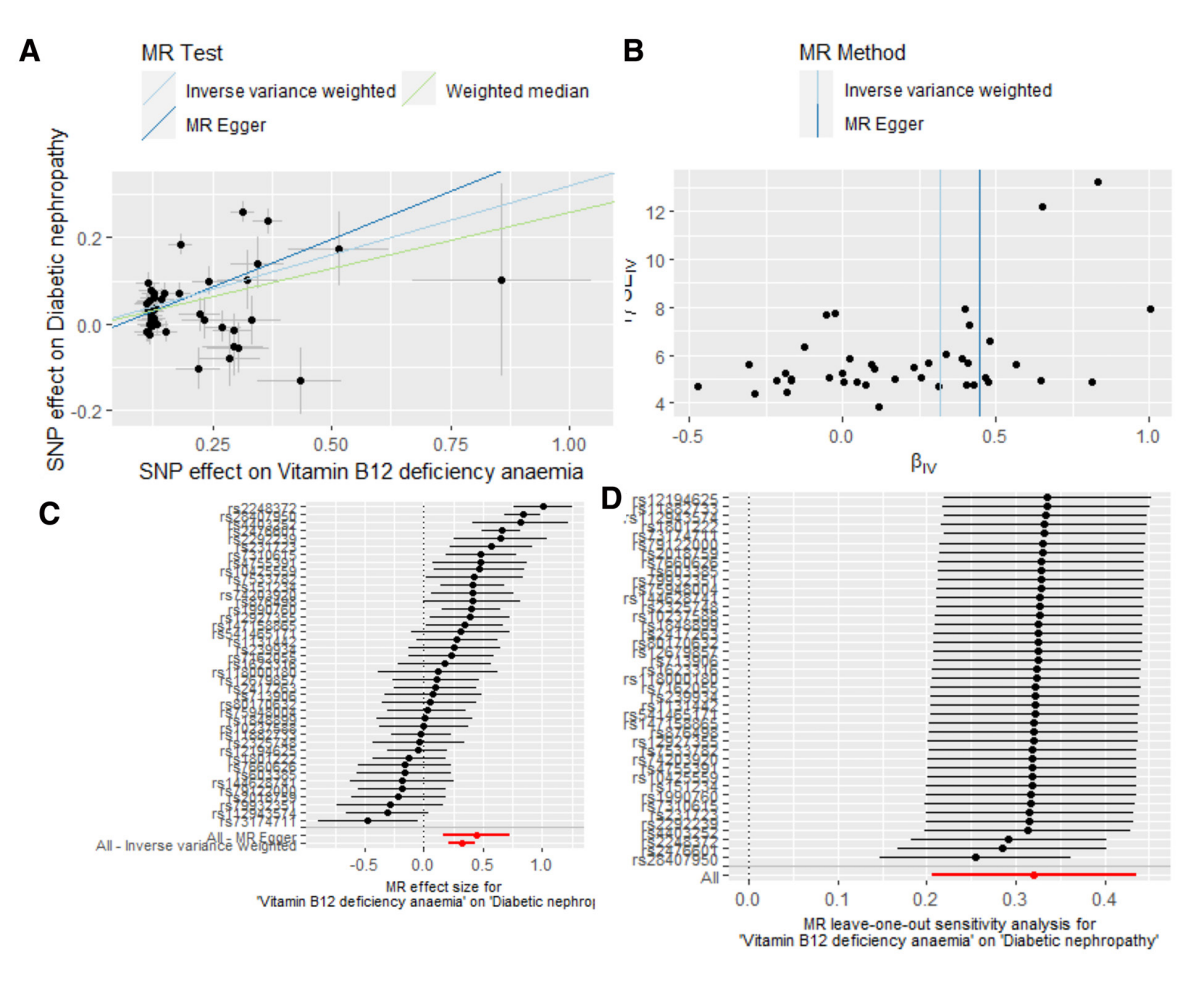
Mendelian randomization analyses of the association between vitamin B12 deficiency anemia and DKD. (A) Scatter plot showing the SNP-specific associations and the estimated effects obtained using the inverse-variance weighted, MR-Egger, and weighted-median methods. (B) Funnel plot used to assess asymmetry among the SNP-specific estimates. (C) Forest plot showing the SNP-specific estimates and 95% confidence intervals, together with the pooled MR-Egger and inverse-variance weighted estimates. (D) Leave-one-out analysis used to assess whether any single SNP disproportionately influenced the pooled estimate. DKD = diabetic kidney disease, IVW = inverse-variance weighted, MR = Mendelian randomization, SE = standard error, SNP= single-nucleotide polymorphism.

After selection, we included 27, 24, and 42 SNPs that were significantly associated with general anemia, iron deficiency anemia, and vitamin B_12_ deficiency anemia, respectively (Tables S1, Supplemental Digital Content 1, S2, Supplemental Digital Content 2, and S3, Supplemental Digital Content 3). The scatter plots (Figs. 6A, 7A, and 8A) and forest plots (Figs. 6C, 7C, and 8C) showed positive MR estimates for the associations of general anemia, iron deficiency anemia, and vitamin B12 deficiency anemia with DKD. The MR estimates for the associations between anemia-related phenotypes and DKD are shown in Figure 5. The IVW estimate revealed that general anemia (IVW: OR = 2.66, 95% CI: 1.53–4.61, *P* = .001), iron deficiency anemia (IVW: OR = 1.57, 95% CI: 1.12–2.19, *P *= .008), and vitamin B_12_ deficiency anemia (IVW: OR = 1.38, 95% CI: 1.23–1.55, *P *= .001) were associated with higher odds of DKD (Table S4, Supplemental Digital Content 4 and Fig. 5). Cochran *Q* tests indicated significant heterogeneity in the analyses of general anemia, iron deficiency anemia, and vitamin B12 deficiency anemia in relation to DKD (Table S5, Supplemental Digital Content 5, all Cochran *Q P* < .05). The MR-Egger intercept tests did not provide statistical evidence of directional horizontal pleiotropy (all *P* > .05); however, nonsignificant intercepts do not exclude other forms of pleiotropy (Figs. 6B, 7B, and 8B). Leave-one-out analyses showed that no single SNP materially influenced the pooled MR estimates (Figs. 6D, 7D, and 8D). The Steiger tests supported the hypothesized direction from anemia-related phenotypes to DKD (all *P* < .05), although they did not eliminate the possibility of bias due to weak instruments, heterogeneity, or pleiotropy (Table S6, Supplemental Digital Content 6). Overall, these findings provide suggestive, rather than definitive, evidence of potential causal associations between anemia-related phenotypes and DKD.

## 4. Discussion

Among patients with CKD, those with DKD have been reported to develop anemia earlier and to have a higher prevalence of anemia than those with nondiabetic kidney disease; anemia in DKD may also be more severe and difficult to correct.^[3]^ Patients with DKD and anemia have also been reported to experience a faster decline in kidney function and a shorter time to dialysis initiation than those without anemia.^[20]^ Some studies have reported that anemia may be present before clinically apparent DKD; however, this observation does not exclude the possibility that subclinical kidney dysfunction contributes to anemia.^[21]^ In the present study, anemia was more prevalent among participants with presumed DKD than among participants with diabetes but without presumed DKD (21.6% vs 9.6%).

Because the NHANES component was cross-sectional, the temporal sequence between anemia and presumed DKD could not be established. DKD may itself contribute to anemia through reduced erythropoietin production, chronic inflammation, impaired iron utilization, and nutritional deficiencies. Therefore, the observed association may be bidirectional, and the NHANES findings should not be interpreted as demonstrating that anemia causes DKD.

Using data from the 2009–2018 NHANES cycles, we evaluated the associations of anemia and Hb levels with the odds of presumed DKD among adults with type 2 diabetes. The RCS analyses indicated nonlinear associations between Hb levels and the odds of presumed DKD, albuminuria, and reduced eGFR. The estimated inflection points for the association between Hb levels and presumed DKD were approximately 12.5 g/dL in men and 11.7 g/dL in women. Below these values, increasing Hb levels were associated with a marked decrease in the estimated odds of presumed DKD, whereas above these values, the decline became less pronounced. The corresponding inflection points for reduced eGFR were approximately 11.1 g/dL in men and 8.9 g/dL in women. Below these values, higher Hb levels were associated with lower odds of reduced eGFR, whereas the association gradually attenuated above these values. Similarly, the estimated inflection points for UACR ≥ 30 mg/g were approximately 11.0 g/dL in men and 9.9 g/dL in women. Below these values, the estimated odds of albuminuria decreased markedly with increasing Hb levels, whereas the decline became less pronounced above these values. Participants with presumed DKD had lower serum iron levels and higher mean corpuscular volume than those without presumed DKD.

The mechanisms underlying the high prevalence of anemia among people with diabetes are likely multifactorial. Potential contributing factors include kidney dysfunction, chronic inflammation, nutritional deficiencies, coexisting autoimmune diseases, medication use, and hormonal abnormalities.^[4]^ Our findings were consistent with previous reports showing associations of anemia with reduced eGFR and albuminuria among people with diabetes.^[22-24]^ Iron deficiency is prevalent in patients with CKD.^[25]^ Deficiencies in iron and erythropoietin (EPO) and a reduced response to EPO are potential mechanisms. Abnormalities in diet or iron absorption may contribute to iron deficiency in diabetes patients.^[26,27]^ Mendelian randomization analysis provides a complementary approach for evaluating potential causal associations under specific instrumental-variable assumptions. In the present study, 2-sample MR analysis was used to explore potential causal associations between genetically predicted anemia-related phenotypes and DKD. Our MR analyses provided suggestive, rather than definitive, evidence of potential causal associations of general anemia, iron deficiency anemia, and vitamin B12 deficiency anemia with DKD.

Because only a limited number of independent variants reached the conventional genome-wide significance threshold of *P* < 5 × 10^−8^, a relaxed threshold of *P* < 5 × 10^−6^ was used to increase instrument availability. However, the use of a relaxed significance threshold may increase susceptibility to weak-instrument bias and false-positive findings. Therefore, the MR estimates should be considered exploratory and interpreted cautiously, and further validation using stronger instruments and independent GWAS datasets is warranted.

Significant Cochran *Q* statistics indicated substantial variability among the SNP-specific estimates. This heterogeneity may reflect differences in instrument validity, phenotype heterogeneity, residual horizontal pleiotropy, or other violations of the instrumental-variable assumptions. Although the MR-Egger intercept tests did not provide statistical evidence of directional horizontal pleiotropy and the leave-one-out analyses did not identify a single influential SNP, these analyses have limited statistical power and do not completely exclude pleiotropy. Accordingly, the pooled MR estimates should be interpreted cautiously.

The occurrence of vitamin B12 deficiency in people with diabetes may be related to metformin use, as metformin-associated cobalamin deficiency may result from impaired intestinal absorption and other gastrointestinal mechanisms.^[28,29]^ The prevalence of iron deficiency varies according to the characteristics of the study population. Previous studies have reported depleted iron stores in approximately 43% of patients with anemia and impaired iron utilization in approximately 58%. These abnormalities may be particularly common among women and individuals with impaired kidney function.^[24]^ In another study of patients without kidney disease, none were iron deficient.^[30]^ In the present study, serum iron levels were lower in participants with diabetes and anemia than in nonanemic patients.

Previous studies have associated anemia with adverse clinical outcomes among people with diabetes.^[31,32]^ Anemia has been reported to precede clinically recognized DKD and to be associated with faster kidney-function decline and cardiovascular complications; however, these observational findings do not by themselves establish causality.^[33]^ These findings support greater clinical attention to anemia in patients with diabetes and kidney disease. Hemoglobin assessment may help identify patients who warrant further evaluation for renal impairment; however, whether correction of anemia delays progression to ESRD or reduces mortality requires confirmation in prospective interventional studies.

The strength of our study lies in the combination of a large-sample observational study based on the NHANES with a 2-sample MR analysis. The NHANES analysis allowed us to examine the associations of anemia and Hb levels with the odds of presumed DKD from a population-based epidemiological perspective, whereas MR analysis may reduce susceptibility to conventional confounding and reverse causation, provided that the instrumental-variable assumptions are satisfied. Nevertheless, MR remains vulnerable to weak-instrument bias, horizontal pleiotropy, phenotype heterogeneity, and other violations of these assumptions. The generally concordant direction of the observational and MR estimates strengthens the coherence of the findings; however, concordance between the 2 approaches does not by itself establish causality.

However, this study also has limitations. First, the NHANES analysis was cross-sectional and, therefore, could not establish temporality or exclude reverse causation. Second, presumed DKD was defined using a single measurement of UACR and eGFR among participants with diabetes. The persistence of kidney abnormalities for at least 3 months could not be confirmed, and kidney disease attributable to nondiabetic causes could not be fully excluded, which may have resulted in outcome misclassification. Third, the complete-case analysis may have introduced selection bias, and residual confounding may remain despite multivariable adjustment. Fourth, the relaxed SNP-selection threshold used in the MR analysis may have increased susceptibility to weak-instrument bias and false-positive findings. Fifth, significant heterogeneity was observed in the MR analyses, and the absence of a statistically significant MR-Egger intercept does not exclude all forms of horizontal pleiotropy. Finally, the observational analysis was based on a multiethnic population in the United States, whereas the GWAS datasets were derived predominantly from individuals of European ancestry. This difference may limit the comparability and generalizability of the findings to other populations.

## 5. Conclusion

In conclusion, anemia was associated with higher odds of presumed DKD, albuminuria, and reduced eGFR among adults with type 2 diabetes in the NHANES population. Two-sample Mendelian randomization analyses provided suggestive, rather than definitive, evidence that genetically predicted general anemia, iron deficiency anemia, and vitamin B12 deficiency anemia may be associated with DKD. However, the cross-sectional design, potential outcome misclassification, relaxed SNP-selection threshold, and significant heterogeneity limit definitive causal inference. Hemoglobin assessment may help identify patients with diabetes who warrant further evaluation for kidney disease. Prospective studies and independent Mendelian randomization analyses are needed to confirm the direction and magnitude of these associations.

## Acknowledgments

We thank the NHANES research team, the investigators who made the GWAS summary data publicly available, and all participants who contributed to these studies.

## Author contributions

**Conceptualization:** Xi Sun, Huanjing Wang.

**Data curation:** Zhanzheng Wang, Yanyun Ren.

**Funding acquisition:** Xi Sun.

**Investigation:** Xi Sun, Huanjing Wang, Yanyun Ren.

**Writing – original draft:** Xi Sun, Huanjing Wang, Zhanzheng Wang, Yanyun Ren.

**Writing – review & editing:** Xi Sun.













## References

[1] Eriksson D Goldsmith D Teitsson S Jackson J van Nooten F. Cross-sectional survey in CKD patients across Europe describing the association between quality of life and anaemia. BMC Nephrol. 2016;17:97. 27460779 10.1186/s12882-016-0312-9 PMC4962379

[2] Thorp ML Johnson ES Yang X Petrik AF Platt R Smith DH. Effect of anaemia on mortality, cardiovascular hospitalizations and end‐stage renal disease among patients with chronic kidney disease. Nephrology (Carlton). 2009;14:240–6. 19207866 10.1111/j.1440-1797.2008.01065.x

[3] Ito K Yokota S Watanabe M. Anemia in diabetic patients reflects severe tubulointerstitial injury and aids in clinically predicting a diagnosis of diabetic nephropathy. Intern Med. 2021;60:1349–57. 33250462 10.2169/internalmedicine.5455-20 PMC8170246

[4] Shams N Osmani MH. Newly diagnosed anemia in admitted diabetics, frequency, etiology and associated factors. J Coll Physicians Surg Pak. 2015;25:242–6. 25899186

[5] Miulescu RD Neamţu MC Margină D Poiană C Păun DL. Associations between thyroid dysfunction and chronic kidney disease. Rom J Diabetes Nutr Metab Dis. 2014;21:37–42.

[6] Basu G Mohapatra A. Interactions between thyroid disorders and kidney disease. Indian J Endocrinol Metab. 2012;16:204–13. 22470856 10.4103/2230-8210.93737 PMC3313737

[7] Astor BC Muntner P Levin A Eustace JA Coresh J. Association of kidney function with anemia: the Third National Health and Nutrition Examination Survey (1988–1994). Arch Intern Med. 2002;162:1401–8. 12076240 10.1001/archinte.162.12.1401

[8] Camargo JL Gross JL. Conditions associated with very low values of glycohaemoglobin measured by an HPLC method. J Clin Pathol. 2004;57:346–9. 15047733 10.1136/jcp.2002.007088 PMC1770269

[9] Keane WF Brenner BM de Zeeuw D.; RENAAL Study Investigators. The risk of developing end-stage renal disease in patients with type 2 diabetes and nephropathy: the RENAAL study. Kidney Int. 2003;63:1499–507. 12631367 10.1046/j.1523-1755.2003.00885.x

[10] Okada A Yamaguchi S Imaizumi T. Modification effects of albuminuria on the association between kidney function and development of anemia in diabetes. J Clin Endocrinol Metab. 2024;109:1012–32. 37955878 10.1210/clinem/dgad660 PMC10940265

[11] Burgess S Davey Smith G Davies NM. Guidelines for performing Mendelian randomization investigations: update for summer 2023. Wellcome Open Res. 2019;4:186. 32760811 10.12688/wellcomeopenres.15555.1 PMC7384151

[12] Davies NM Holmes MV Davey Smith G. Reading Mendelian randomisation studies: a guide, glossary, and checklist for clinicians. BMJ. 2018;362:k601. 30002074 10.1136/bmj.k601 PMC6041728

[13] Zipf G Chiappa M Porter KS Ostchega Y Lewis BG Dostal J. National health and nutrition examination survey: plan and operations, 1999-2010. Vital Health Stat 1. 2013:1–37. 25078429

[14] Ma R Xie C Wang S Xiao X. Retinol intake is associated with the risk of chronic kidney disease in individuals with type 2 diabetes mellitus: results from NHANES. Sci Rep. 2023;13:11567. 37463986 10.1038/s41598-023-38582-z PMC10354112

[15] Stanton RC. Clinical challenges in diagnosis and management of diabetic kidney disease. Am J Kidney Dis. 2014;63:S3–21. 24461728 10.1053/j.ajkd.2013.10.050

[16] Kim H Hur M Lee S Lee GH Moon HW Yun YM. European Kidney Function Consortium Equation vs. Chronic Kidney Disease Epidemiology Collaboration (CKD-EPI) refit equations for estimating glomerular filtration rate: comparison with CKD-EPI equations in the Korean population. J Clin Med. 2022;11:4323. 35893414 10.3390/jcm11154323 PMC9331398

[17] World Health Organization. Haemoglobin Concentrations for the Diagnosis of Anaemia and Assessment of Severity. Geneva: World Health Organization; 2011. (WHO/NMH/NHD/MNM/11.1).

[18] Johnson CL Paulose-Ram R Ogden CL. National health and nutrition examination survey: analytic guidelines, 1999-2010. Vital Health Stat 2. 2013:1–24. 25090154

[19] Boef AG Dekkers OM le Cessie S. Mendelian randomization studies: a review of the approaches used and the quality of reporting. Int J Epidemiol. 2015;44:496–511. 25953784 10.1093/ije/dyv071

[20] Kim SH Lee KA Jin HY Baek HS Park TS. The relationship between anemia and the initiation of dialysis in patients with type 2 diabetic nephropathy. Diabetes Metab J. 2015;39:240–6. 26124994 10.4093/dmj.2015.39.3.240 PMC4483609

[21] Alicic RZ Tuttle KR. Management of the diabetic patient with advanced chronic kidney disease. Semin Dial. 2010;23:140–7. 20374553 10.1111/j.1525-139X.2010.00700.x

[22] Wolf G Müller N Hunger-Battefeld W Kloos C Müller UA. Hemoglobin concentrations are closely linked to renal function in patients with type 1 or 2 diabetes mellitus. Kidney Blood Press Res. 2008;31:313–21. 18791327 10.1159/000155230

[23] Bulum T Prkacin I Blaslov K Zibar K Duvnjak L. Association between red blood cell count and renal function exist in type 1 diabetic patients in the absence of nephropathy. Coll Antropol. 2013;37:777–82. 24308216

[24] Thomas MC MacIsaac RJ Tsalamandris C Power D Jerums G. Unrecognized anemia in patients with diabetes: a cross-sectional survey. Diabetes Care. 2003;26:1164–9. 12663591 10.2337/diacare.26.4.1164

[25] Besarab A Coyne DW. Iron supplementation to treat anemia in patients with chronic kidney disease. Nat Rev Nephrol. 2010;6:699–710. 20956992 10.1038/nrneph.2010.139

[26] Mcgill JB Bell DSH. Anemia and the role of erythropoietin in diabetes. J Diabetes Complications. 2006;20:262–72. 16798479 10.1016/j.jdiacomp.2005.08.001

[27] Bosman DR Winkler AS Marsden JT Macdougall IC Watkins PJ. Anemia with erythropoietin deficiency occurs early in diabetic nephropathy. Diabetes Care. 2001;24:495–9. 11289474 10.2337/diacare.24.3.495

[28] Ahmed MA. Metformin and vitamin B12 deficiency: where do we stand? J Pharm Pharm Sci. 2016;19:382–98. 27806244 10.18433/J3PK7P

[29] Infante M Leoni M Caprio M Fabbri A. Long-term metformin therapy and vitamin B12 deficiency: an association to bear in mind. World J Diabetes. 2021;12:916–31. 34326945 10.4239/wjd.v12.i7.916 PMC8311483

[30] Craig KJ Williams JD Riley SG. Anemia and diabetes in the absence of nephropathy. Diabetes Care. 2005;28:1118–23. 15855576 10.2337/diacare.28.5.1118

[31] Vlagopoulos PT Tighiouart H Weiner DE. Anemia as a risk factor for cardiovascular disease and all-cause mortality in diabetes: the impact of chronic kidney disease. J Am Soc Nephrol. 2005;16:3403–10. 16162813 10.1681/ASN.2005030226

[32] Song HY Wei CM Zhou WX Hu HF Wan QJ. Association between admission hemoglobin level and prognosis in patients with type 2 diabetes mellitus. World J Diabetes. 2021;12:1917–27. 34888016 10.4239/wjd.v12.i11.1917 PMC8613662

[33] Toft G Heide-Jørgensen U van Haalen H. Anemia and clinical outcomes in patients with non-dialysis dependent or dialysis dependent severe chronic kidney disease: a Danish population-based study. J Nephrol. 2020;33:147–56. 31587136 10.1007/s40620-019-00652-9 PMC7007417

